# Extreme Learning Machine-Based Classification of ADHD Using Brain Structural MRI Data

**DOI:** 10.1371/journal.pone.0079476

**Published:** 2013-11-19

**Authors:** Xiaolong Peng, Pan Lin, Tongsheng Zhang, Jue Wang

**Affiliations:** 1 The Key Laboratory of Biomedical Information Engineering of the Ministry of Education, Biomedical Engineering Institute, School of Life Science and Technology, Xi’an Jiaotong University, Xi’an, People’s Republic of China; 2 National Engineering Research Center of Health Care and Medical Devices, Xi’an Jiaotong University Branch, Xi’an, People’s Republic of China; 3 Department of Neurology, University of New Mexico, Albuquerque, New Mexico, United States of America; College of Mechatronics and Automation, National University of Defense Technology, China

## Abstract

**Background:**

Effective and accurate diagnosis of attention-deficit/hyperactivity disorder (ADHD) is currently of significant interest. ADHD has been associated with multiple cortical features from structural MRI data. However, most existing learning algorithms for ADHD identification contain obvious defects, such as time-consuming training, parameters selection, etc. The aims of this study were as follows: (1) Propose an ADHD classification model using the extreme learning machine (ELM) algorithm for automatic, efficient and objective clinical ADHD diagnosis. (2) Assess the computational efficiency and the effect of sample size on both ELM and support vector machine (SVM) methods and analyze which brain segments are involved in ADHD.

**Methods:**

High-resolution three-dimensional MR images were acquired from 55 ADHD subjects and 55 healthy controls. Multiple brain measures (cortical thickness, etc.) were calculated using a fully automated procedure in the FreeSurfer software package. In total, 340 cortical features were automatically extracted from 68 brain segments with 5 basic cortical features. F-score and SFS methods were adopted to select the optimal features for ADHD classification. Both ELM and SVM were evaluated for classification accuracy using leave-one-out cross-validation.

**Results:**

We achieved ADHD prediction accuracies of 90.18% for ELM using eleven combined features, 84.73% for SVM-Linear and 86.55% for SVM-RBF. Our results show that ELM has better computational efficiency and is more robust as sample size changes than is SVM for ADHD classification. The most pronounced differences between ADHD and healthy subjects were observed in the frontal lobe, temporal lobe, occipital lobe and insular.

**Conclusion:**

Our ELM-based algorithm for ADHD diagnosis performs considerably better than the traditional SVM algorithm. This result suggests that ELM may be used for the clinical diagnosis of ADHD and the investigation of different brain diseases.

## Introduction

Attention-deficit/hyperactivity disorder (ADHD) is one of the most prevalent behavioral disorders in childhood and adolescence. Approximately 5% of school-age children and 2–4% of adults are diagnosed with ADHD or have ADHD-associated symptoms [1]. ADHD is typically characterized by inattention, hyperactivity, impulsivity and impaired executive function, and its diagnosis is normally made on the basis of these behavioral symptoms. However, there is currently no diagnostic laboratory test for ADHD. ADHD diagnosis may include psychological tests, such as the ADHD Rating Scale (ADHD-RS), Conners Parent Rating Scale and Brown Attention Deficit Disorder Scale (BADDS). The efficiency of the diagnostic process is generally low because testing requires a long, tedious clinical interview. In addition, traditional ADHD diagnosis methods commonly lead to misdiagnosis. For instance, approximately 20% of children are misdiagnosed because they are younger than their classmates [2], [3]. Therefore, a rapid, accurate and objective diagnostic tool is needed to improve the understanding, prevention and treatment of ADHD.

To aid the development of a new ADHD diagnostic method, objective experimental differences between ADHD and control subjects (CS) should be defined. To date, most studies have explored differences in the connectivity of complex human brain networks between ADHD and normal children [4]–[8]. Most of these studies employ electroencephalographic (EEG) or magnetoencephalographic (MEG) detection technology to record electromagnetic brain activity. However, these recordings are subject to electromagnetic interference from the external environment, such as 50 Hz power-line interference, or signal reductions by the human skull [9]–[12]. Structural imaging tools, such as magnetic resonance imaging (MRI) and functional MRI, have been extensively utilized to study the anatomical aspects of human brain disorders and to identify the fundamental differences between ADHD and normal subjects [13]–[16]. Additionally, brain imaging technologies have also been applied to the ADHD diagnosis and classification. In the early days, researchers use single-photon emission computed tomography (SPECT) to compare the pattern of regional cerebral perfusion in groups of children with ADHD during a computerized performance test [17]. With the development of imaging techniques, a growing number of noninvasive imaging technologies begin to be applied in ADHD classification, especially two particularly prominent kinds of imaging methods: morphological information based on brain MRI data and brain connectivity based on functional MRI [18], [19].

In the past several years, numerous anatomic imaging studies have accrued evidence for structural brain abnormalities in ADHD. Results for children with ADHD from recent findings showed a decrease in total cortical volume of over 7 and 8% and a decrease in surface area of over 7% bilaterally [20]. Anatomical abnormalities have also been observed in cortical thickness and folding, especially in posterior brain regions and anterior brain regions, including left/right superior temporal and parietal lobes, temporoparietal junction, and insula [21], [22]. All these abnormalities in ADHD suggest that structural MRI data of human brain should be a kind of ideal classification feature for ADHD diagnosis.

Moreover, structural MRI has a high resolution and uses relatively stable imaging technology. Several studies using structural MRI have demonstrated anatomical differences between ADHD and normal children [23]–[25]. Anatomical MRI showed that the maturation of cortical thickness and the surface area developmental trajectory of the right prefrontal cortex is delayed in ADHD children relative to typically developing children [7]. Additionally, machine pattern recognition techniques based on structural MRI data have been extensively applied to diagnose many diseases. For example, brain tumor volume can be obtained from structural MRI data using computer-aided diagnosis [26], [27]. Outstanding Alzheimer’s disease (AD) classification accuracy has been achieved using whole-brain anatomical MRI with SVM, which can aid early AD diagnosis [28]–[31]. These successful examples of brain disease diagnosis prompted us to develop a method that combines brain morphological MRI with a learning machine method, which may be used to supplement existing cognitive batteries during diagnostic procedures.

To date, traditional machine learning techniques have been utilized to distinguish the MRI data of two groups of subjects who have multiple obvious defects. This involves time-consuming training sessions for the experimental dataset, classification inefficiency with changes in sample size and selection of one or more parameters for the classifier [29], [30]. For example, when classifying mild cognitive impairment subtypes using a support vector machine, Haller and colleagues had to iteratively explore the parameter gamma from 0.01 to 0.09 [32]. In addition, the testing accuracy is not always satisfactory enough for practical classification applications [31].

In this study, we focused on developing an automatic, effective, rapid and accurate ADHD diagnosis method to overcome the deficiencies of traditional methods. We first proposed an ADHD classification model using the extreme learning machine (ELM) with F-score and SFS feature selection methods to provide objective clinical diagnosis. The simple and efficient ELM method was introduced to build a robust model for ADHD classification. It is based on 5 basic cortical properties: thickness, surface area, folding index, curvature and volume. Our findings demonstrate that the ELM learning model performs better and has an extraordinarily higher accuracy than the commonly used SVM learning algorithm in terms of computing efficiency and the dependence of experimental dataset size. We also found that the surface area (SA) and volume (V) data of the human brain provide the most salient information for discriminating between ADHD and CS.

## Materials and Methods

### 1. Subjects

The data used in the present study were part of the dataset from the Peking University (Peking_1 and Peking_2) ADHD-200 Global Competition Test Dataset (http://fcon_1000.projects.nitrc.org/indi/adhd200/). The dataset contains a total of 152 subjects including 59 ADHD and 93 healthy controls. Fifty-five of 59 ADHD subjects with were selected for the current study according to the age range from 9 to 14 (mean age 11.8) and 4 overage subjects were excluded. Other fifty-five of 93 age matched healthy adolescents were selected to form the control group (mean age 11.5). Patients with a history of medication use were also included. The inclusion criteria were as follows: 1) right-handedness; 2) no lifetime history of head trauma with loss of consciousness; 3) no history of neurological disease, and no diagnosis of schizophrenia, affective disorder, pervasive development disorder, or substance abuse and 4) full-scale Wechsler Intelligence Scale for Chinese Children-Revised (WISCC-R) score of greater than 80.

### 2. MRI

MRI data were downloaded from the ADHD-200 Global Competition website (http://fcon_1000.projects.nitrc.org/indi/adhd200/). A description of the Peking University ADHD-200 Global Competition data acquisition can be found in the scan parameters item of the website. Briefly, the MRI data were collected using a SIEMENS TRIO 3-Tesla scanner. The MRI protocol included acquiring a high-resolution T1-weighted MPRAGE volume (voxel size 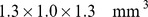) using a custom pulse sequence with the following parameters: 2530/3.39 ms (TR/TE) and 1.33 mm (slice thickness).

### 3. MRI Data Processing

**Figure 1:**
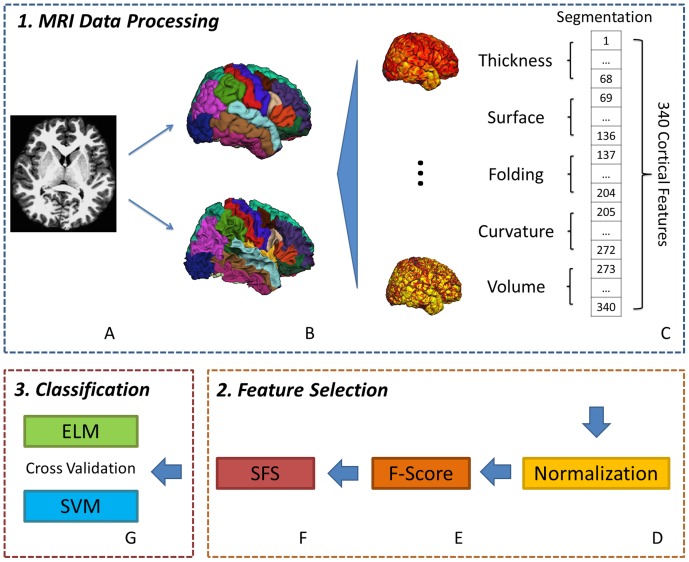
A flowchart for ADHD classification using human cortical feature measurements from MRI. (A) A T1-weighted anatomical image preprocessed with nonuniformity correction and registration. (B) The upper and lower images refer to the pial vertices (outer gray surface) and white vertices (inner gray surface), respectively, that were extracted and reconstructed in stereotaxic space from (A). (C) Five basic cortical features, including thickness, surface area, folding index, curvature and volume, were measured from the divisional cortical surfaces, comprising a total of 340 brain features for each subject. (D) All the brain features were normalized to the range from 0 to 1. (E) The normalized data were rearranged in accordance with the F-score in descending order. (F) The SFS method was used to further select the features that enhance the classification accuracy. (G) The classification accuracy of both ELM and SVM learning algorithms was tested using the leave-one-out cross-validation method.

The FreeSurfer 5.10 software package was utilized for cortical reconstruction and volumetric segmentation (FreeSurfer v5.10, http://surfer.nmr.mgh.harvard.edu/fswiki). For processing, the original MRI data were first subjected to a series of preprocessing steps, including motion correction, T1-weighted image averaging, registration of the volume to Talairach space and stripping the skull with a deformable template model (Figure 1A). By encoding the shape of the corpus callosum and pons in the Talairach space and following the intensity gradients from the white matter to the cerebrospinal fluid, the white surface and the pial surface were generated for each hemisphere (Figure 1B). Once these surfaces were known, a cortical surface-based atlas was mapped to a sphere aligning the cortical folding patterns, which provided accurate matching of the morphologically homologous cortical locations across subjects. The average shortest distance between white and pial surfaces denoted the cortical thickness at each vertex of the cortex. Surface area was calculated by computing the area of every triangle in a standardized spherical surface tessellation. The local curvature was computed using the registration surface based on the folding patterns. The folding index over the whole cortical surface was measured using the method developed by Schaer. In the present study, the FreeSurfer pipeline was used to automatically generate the five basic cortical features. Each basic feature was divided into 68 components based on brain segments, which comprise a total of 340 cortical features for each subject (Figure 1C). The indexes of 340 cortical features are briefly presented in Table 1.

**Table 1:** Information for the experimental dataset.

Feature Number	Basic Features	Index of Segmentations
Feature 1	Cortical Thickness	1–68
Feature 2	Surface Area	69–136
Feature 3	Volume	137–204
Feature 4	Folding Index	205–272
Feature 5	Intrinsic Curvature	273–340

### 4. Feature Selection

After normalizing all the brain features data to the range from 0 to 1 (Figure 1D), we utilized the F-score method (Figure 1E) and the sequential forward selection (SFS) method (Figure 1F) for feature optimization selection of the 340 cortical features to achieve a high classification accuracy. We then set the selected features as the experimental dataset for ADHD classification. The basic principles of these two feature selection methods are briefly described below.

#### 4.1. F-Score

F-score (Fisher score) is a simple and efficient feature selection criterion obtained by measuring the discrimination between two sets of real numbers [33]. Given training vectors 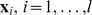, the F-score of the 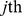 feature is defined as
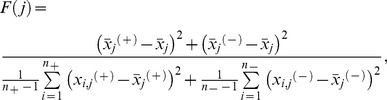
(1)where 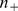 and 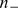 are the number of positive and negative instances, respectively, 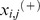 and 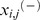 are the 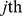 feature of the positive and negative instances, respectively, and 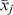, 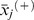 and 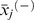 are the averages of the whole, positive and negative datasets, respectively. A larger F-score indicates that the feature is more significant because the numerator refers to the variance between two classes and the denominator denotes the variance within each class.

#### 4.2. SFS

Sequential forward selection (SFS) is a simple efficient feature selection approach [34]. A subset was defined by iteratively adding one feature at a time to an empty set to achieve the maximum intermediate criterion value. Then, the subset of 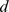 features was generated using the SFS method:
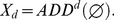
(2)


### 5. Classification

As shown in Figure 1G, both the SVM and ELM classifiers were used for the experimental dataset of 110 subjects to perform the leave-one-out cross-validation. Validation involves using features of a single subject from the whole experimental dataset for testing and using the remaining subjects to train the classifier. This processing is repeated for all the subjects. We then evaluated the ADHD classification efficiency of both learning algorithms by comparing their average testing accuracy and classification time. The descriptions of these two learning algorithms are shown below.

#### 5.1. SVM learning algorithm

Support vector machines (SVM) are popular machine learning methods for classification and regression that are based on the learning theory originally developed by Vapnik and his colleagues in 1995 [35]. In SVM, an n-class problem is converted into n two-class problems. For each two-class problem, the original 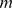-dimensional input vector 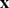 is mapped into the 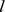-dimensional (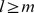) dot product space (feature space) using a nonlinear vector function to enhance linear separability. In this high-dimensional feature space, the optimal separating hyperplane that has the maximal margin to the nearest training datum needs to be found. Once processing is completed, the testing data can also be mapped into the feature space, and then a class is assigned to the testing data.

In the present study, the LIBSVM software package was applied to implement the SVM algorithm, and simple efficient linear function and radial basis function (RBF) were respectively selected as the kernel functions. LIBSVM, an integrated software package that is extensively used for regression and classification in machine learning, was developed by Dr. Chih-Jen Lin and his colleagues (LIBSVM v3.12 available at http://www.csie.ntu.edu.tw/~cjlin/libsvm/).

#### 5.2. ELM learning algorithm

Extreme learning machine (ELM) is an extremely fast learning algorithm with good generalization performance that was developed by Huang and his research group [36]. Traditional single hidden-layer feedforward neural networks (SLFNs), such as the back propagation (BP) learning algorithm, have been extensively used for research in many fields. These methods may require a search for the specific input weights and hidden layer biases to minimize the cost function, which usually makes it difficult to keep the computing speed and classification accuracy within an acceptable range. According to Theorem 1 and Theorem 2 shown in the Appendix S1, the input weight 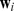 and the hidden layer biases 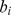 of SLFNs for ELM can be randomly assigned if the activation functions in the hidden layer are infinitely differentiable [37], [38]. Therefore, training an SLFN is equivalent to finding a least squares solution 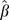 of the linear system 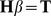:
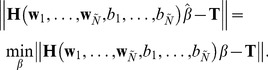
(3)


However, for most cases the number of hidden nodes is far less than the number of distinct training samples 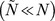, which means 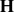 is not a square matrix, and there may not exist 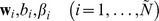 such that 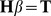. According to Theorem 3, the smallest norm least squares solution of the linear system is
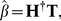
(4)where 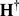 is the Moore-Penrose generalized inverse of matrix 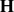. With the completion of the model of the ELM algorithm, the testing data could be efficiently classified.

### 6. Selection of Classification Algorithm Parameters

Our extreme learning machine (ELM) training and classification computing program was compiled using MATLAB based on the relative research theories of Dr. Huang. In this study, we selected a simple sigmoidal kernel function 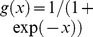 and set the number of hidden nodes to 20. The SVM classification simulations were carried out using the MATLAB interface to the C-coded LIBSVM package developed by Dr. Lin’s team. In our experiments, two kernel parameters 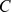 and 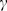 for radial basis function (RBF) SVM and one kernel parameter 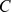 for linear SVM needed to be determined according to the LIBSVM user guidelines. Because the SVM algorithm performs particularly poor on the experimental dataset when the default parameters setting is selected, we used the grid-search method on 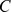 and 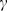 to obtain suitable parameters for the SVM algorithm before the training. A practical method of identifying good parameters involves attempting exponentially growing sequences of 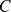 and 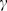. The pair of 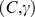 values with the best cross-validation accuracy is selected as the best setting. In the present study, the search scales of these two parameters were set to 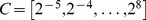 and 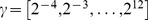. In addition, it is worth noting that, although the grid-search method may improve the classification accuracy of the SVM algorithm, it also significantly increases the total training time of SVM. This will be discussed below in the computational efficiency section.

Additionally, as the threshold for each decision function of the binary method may affect the performance of classification a lot, it should be determined according to the receiver operating characteristics (ROC) curves. In the current study, thresholds of all three algorithms were set to the default 0 since the discrimination showed balance performance between true positive rate and false positive rate then.

### 7. Permutation Tests

The permutation tests have been adapted to assess statistical significance of the classifier and its performance in many research fields [39], [40]. A brief description of permutation tests processing steps is as follows: choosing the statistic of classifier, randomly permuting the class label of the training data before training, performing cross-validation on permuted training set and repeating the procedures as many times as needed. In this study, the generation rate was selected as the statistic and the times of repetition were set to 10000. We hypothesized that the classifier could not learn the relationship between data and labels reliably. The P-value 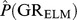 represents the probability of observing a prediction rate no less than 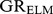 obtained by classifier trained on real labeled data. If the generation rate 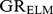 exceeded the 95% confidence interval of training on randomly relabeled data, the null hypothesis was rejected and the classifier learned the relationship with a probability of being wrong of at most 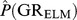.

## Results

### 1. Performance of ELM, SVM-Linear and SVM-RBF in ADHD Classification based on F-score Feature Selection

**Figure 2:**
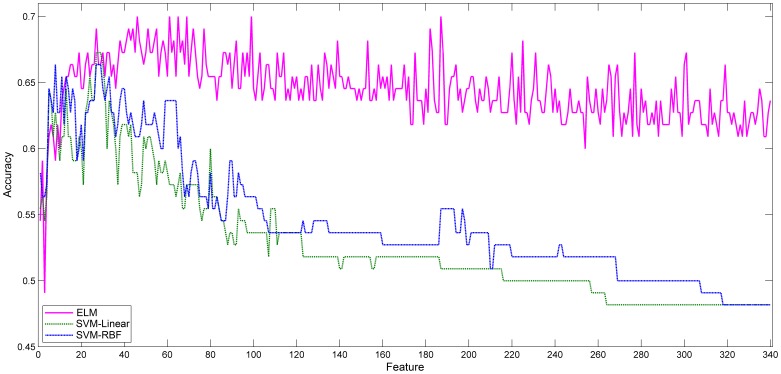
Comparison of the testing accuracy of ELM, SVM-Linear and SVM-RBF in ADHD classification based on F-score feature selection.

The F-score feature ranking method was used to arrange the 340 features of ADHD and CS in descending order according to the F-score value. We combined each feature with all preceding feature rows as an experimental dataset. For example, the seventh feature (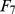) would be combined with the previous six feature (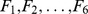) rows to build an experimental dataset defined as the seventh experimental dataset (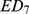). This process was repeated for all the features in sequential order to generate 340 experimental datasets (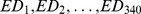). Next, leave-one-out cross-validation was applied to compare the performance of both methods in ADHD classification. The results are shown in Figure 2.

The overall testing accuracy of the ELM algorithm in ADHD classification was significantly higher than that of the both SVM algorithms. Because the high accuracy of these methods depended mainly on previous experimental datasets, we list the detailed results of the first 50 experimental datasets in Table 2. The ELM learning algorithm achieved a maximum classification accuracy of 70% at the forty-sixth experimental dataset (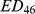). The SVM-Linear and SVM-RBF algorithms respectively reached maximum of 67.27% at the twenty-seventh experimental dataset (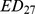) and 66.36% at the eighth experimental dataset (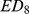). Thus, we concluded that ELM has a better accuracy in ADHD classification than both SVM algorithms.

**Table 2:** Comparison of the training and testing accuracy of ELM and SVM in ADHD classification.

	The composition of ED ( 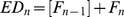 )		Train Acc. (%) ± SD			Test Acc. (%)		
ED No	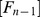	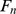 : [Index, Property, Region]	ELM	SVM Linear	SVM RBF	ELM	SVM Linear	SVM RBF
1	–	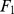 : [271, FI, R-Transversetemporal]	73.08±0.70	55.46±0.46	58.86±0.71	54.55	55.45	58.18
2	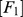	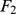 : [114, SA, R-Lingual]	80.33±2.61	60.08±1.00	60.08±1.00	59.09	56.36	56.36
3	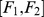	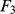 : [80, SA, L-Lingual]	79.10±1.68	59.93±1.10	59.87±1.73	49.09	54.55	56.36
4	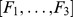	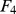 : [225, FI, L-Postcentral]	80.92±2.66	61.57±1.13	60.30±1.15	58.18	56.36	57.27
5	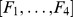	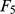 : [106, SA, R-Cuneus]	82.33±1.79	65.02±1.62	68.68±1.38	60.91	64.55	64.55
6	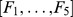	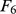 : [209, FI, L-Entorhinal]	81.63±1.62	65.68±1.37	70.08±0.75	61.82	63.64	63.64
7	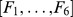	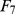 : [72, SA, L-Cuneus]	81.82±1.84	65.49±1.37	96.16±0.48	60.91	60.91	62.73
8	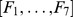	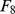 : [224, FI, L-Pericalcarine]	81.06±2.48	65.76±1.24	98.20±0.17	59.09	62.73	**66.36**
9	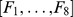	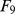 : [220, FI, L-Paracentral]	81.31±2.08	67.11±1.19	96.35±0.37	61.82	60.91	62.73
10	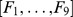	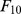 : [265, FI, R-Superiorfrontal]	81.92±2.22	66.46±1.05	97.28±0.21	60.00	59.09	62.73
11	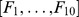	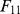 : [227, FI, L-Precentral]	82.60±2.07	68.57±1.62	97.29±0.19	63.64	60.91	65.45
12	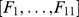	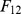 : [272, FI, R-Insula]	81.67±1.94	67.07±1.15	68.64±1.27	64.55	60.91	61.82
13	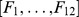	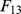 : [252, FI, R-Middletemporal]	82.21±2.21	69.20±0.99	69.56±0.78	65.45	64.55	65.45
14	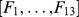	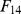 : [81, SA, L-Medialorbitofrontal]	85.37±1.72	70.19±1.83	73.99±1.20	65.45	60.91	64.55
15	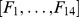	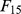 : [148, V, L-Lingual]	85.08±1.83	70.15±1.77	74.06±1.11	66.36	60.91	62.73
16	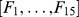	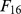 : [122, SA, R-Pericalcarine]	86.18±1.80	70.39±1.72	75.36±0.54	66.36	59.09	64.55
17	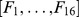	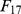 : [182, V, R-Lingual]	86.22±2.21	70.03±1.55	76.16±0.56	65.45	59.09	63.64
18	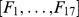	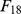 : [69, SA, L-Bankssts]	86.92±2.08	71.74±1.27	71.70±1.31	65.45	59.09	59.09
19	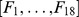	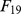 : [174, V, R-Cuneus]	87.51±1.85	71.83±1.26	71.80±1.15	67.27	60.91	60.00
20	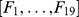	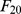 : [88, SA, L-Pericalcarine]	86.18±1.80	70.21±1.25	70.23±1.28	64.55	60.00	61.82
21	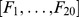	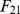 : [210, FI, L-Fusiform]	87.03±2.10	71.27±1.24	73.60±1.30	64.55	57.27	59.09
22	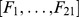	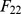 : [207, FI, L-Caudalmiddlefrontal]	87.00±1.98	71.11±0.89	71.38±0.93	66.36	62.73	62.73
23	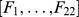	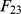 : [87, SA, L-Parstriangularis]	86.19±2.48	74.30±1.20	73.86±1.03	67.27	63.64	62.73
24	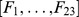	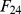 : [204, V, R-Insula]	85.98±2.27	74.66±1.02	73.85±1.00	65.45	65.45	63.64
25	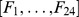	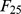 : [208, FI, L-Cuneus]	85.47±2.39	72.85±1.23	73.51±1.15	66.36	63.64	63.64
26	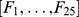	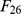 : [256, FI, R-Parsorbitalis]	85.11±2.57	73.03±1.17	73.17±1.11	66.36	63.64	63.64
27	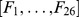	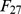 :[240,FI,R-Caudalanteriorcingulate]	85.33±2.46	72.89±0.82	73.34±0.73	69.09	**67.27**	66.36
28	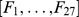	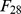 : [264, FI, R-Rostralmiddlefrontal]	84.67±2.27	72.95±0.82	73.37±0.80	66.36	67.27	66.36
29	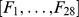	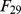 : [140, V, L-Cuneus]	85.43±2.69	72.94±0.81	73.38±0.81	66.36	67.27	66.36
30	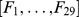	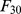 : [249, FI, R-Lateralorbitofrontal]	85.17±2.44	75.84±0.81	75.83±0.92	67.27	66.36	64.55
31	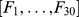	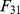 : [255, FI, R-Parsopercularis]	84.97±2.39	75.79±0.88	75.94±0.91	65.45	64.55	63.64
32	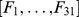	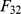 : [89, SA, L-Postcentral]	85.01±2.89	75.06±0.90	78.17±1.14	67.27	60.00	64.55
33	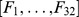	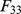 : [218, FI, L-Middletemporal]	85.74±2.39	73.73±0.86	77.70±1.06	67.27	63.64	65.45
34	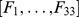	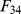 : [119, SA, R-Parsopercularis]	85.69±2.49	73.86±0.91	77.47±0.99	65.45	62.73	62.73
35	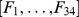	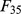 : [232, FI, L-Superiorparietal]	84.76±2.66	73.80±1.01	77.51±1.07	66.36	61.82	62.73
36	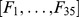	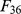 : [101, SA, L-Transversetemporal]	84.38±2.95	73.52±0.98	77.12±1.01	64.55	60.00	60.91
37	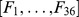	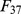 : [136, SA, R-Insula]	84.52±2.75	73.69±0.97	76.76±1.07	66.36	57.27	61.82
38	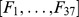	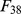 : [84, SA, L-Paracentral]	86.28±3.45	74.70±0.93	79.28±0.96	68.18	60.91	63.64
39	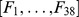	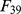 : [169, V, L-Transversetemporal]	85.67±3.38	74.80±0.97	79.13±1.10	67.27	61.82	64.55
40	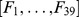	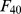 : [130, SA, R-Superiorparietal]	85.22±3.14	77.48±1.19	78.21±1.37	67.27	61.82	64.55
41	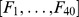	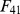 : [131, SA, R-Superiortemporal]	84.45±3.35	77.44±1.09	79.47±1.13	68.18	61.82	62.73
42	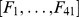	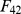 : [190, V, R-Pericalcarine]	85.72±3.31	77.71±1.35	79.22±1.51	69.09	60.91	61.82
43	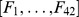	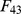 : [238, FI, L-Insula]	84.42±3.32	77.62±1.39	78.77±1.02	68.18	61.82	62.73
44	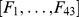	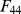 : [74, SA, L-Fusiform]	85.22±3.33	76.43±1.35	74.36±1.17	69.09	58.18	61.82
45	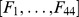	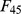 : [262, FI, R-Precuneus]	85.53±3.18	70.05±1.02	74.56±1.07	67.27	58.18	60.91
46	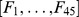	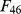 : [258, FI, R-Pericalcarine]	84.87±3.10	76.13±0.98	74.52±0.96	**70.00**	58.18	60.91
47	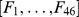	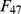 : [102, SA, L-Insula]	84.89±3.02	78.69±1.10	74.73±1.01	68.18	56.36	60.91
48	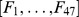	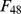 : [73, SA, L-Entorhinal]	84.92±3.32	80.23±0.87	80.69±1.14	67.27	57.27	61.82
49	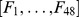	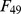 : [105, SA, R-Caudalmiddlefrontal]	85.50±3.57	79.00±1.00	83.29±0.81	66.36	60.91	63.64
50	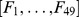	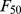 : [133, SA, R-Frontalpole]	85.43±3.27	79.19±0.97	83.38±1.10	67.27	60.00	61.82

ED: Experimental Dataset; SA: Surface Area; V: Volume; FI: Folding Index; L: Left; R: Right.

**Figure 3:**
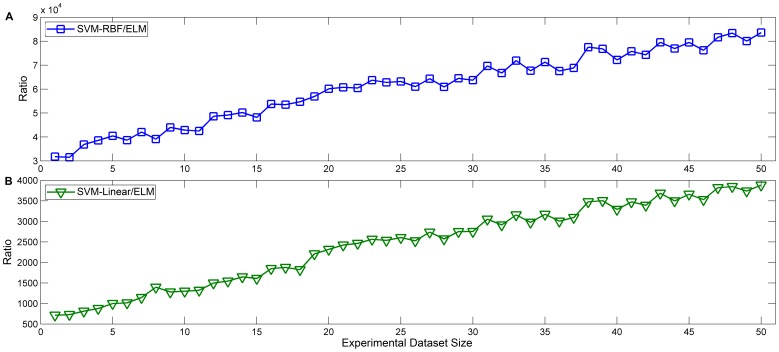
The ratio of SVM grid-search time to ELM training time. (A) The ratio of SVM-RBF grid-search time to ELM training time. (B) The ratio of SVM-Linear grid-search time to ELM training time.

For the SVM algorithm, we considered the grid-search time separately from the SVM training time because it is much longer than the normal training time (more than 1000 times longer). Both ratio of SVM grid-search time to ELM training time for the first 50 experimental datasets increased rapidly with increasing experimental dataset size (Figure 3). This means that the ELM algorithm is much faster at ADHD classification than the SVM algorithm, especially when the experimental dataset is very large.

### 2. ADHD Classification Accuracy Enhancement by SFS

**Figure 4:**
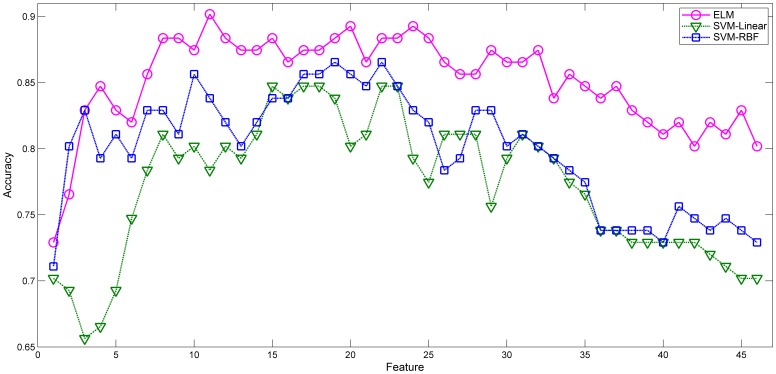
Comparison of the testing accuracy of ELM, SVM-Linear and SVM-RBF in ADHD classification based on SFS feature selection.

The results of ADHD classification show that all three classification algorithms achieve the maximum before the forty-sixth experimental dataset. To further enhance the classification accuracy, the sequential forward selection (SFS) method was executed on the first 46 features of the F-score method and the results are shown in Figure 4.

The testing accuracy of all three methods in ADHD classification were improved as is detailed in Table 3. The ELM algorithm achieved a maximum testing accuracy of 90.18% at the eleventh experimental dataset (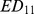), while SVM-Linear and SVM-RBF algorithms respectively reached maximum of 84.73% at the fifteenth experimental dataset (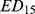) and 86.55% at the nineteenth experimental dataset (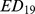). Compared with the traditional SVM classification method, the ELM algorithm performs significantly better than SVM-Linear (paired 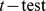, 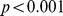) and SVM-RBF (paired 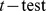, 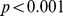).

**Table 3:** Comparison of the training and testing accuracy of ELM and SVM in ADHD classification.

	The composition of ED ( 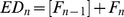 )		Train Acc. ± SD (%)			Test Acc. (%)		
ED No	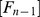	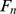 : [Index, Property, Region]	ELM	SVM Linear	SVM RBF	ELM	SVM Linear	SVM RBF
1	–	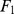 : [72, SA, L-Cuneus]	79.65±1.08	74.27±1.24	76.43±0.68	72.91	70.18	71.09
2	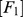	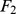 : [271, FI, R-Transversetemporal]	90.62±1.48	75.42±1.15	91.27±0.74	76.55	69.27	80.18
3	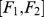	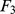 : [238, FI, L-Insula]	95.53±1.57	74.49±0.89	96.18±0.55	82.91	65.64	82.91
4	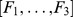	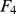 : [140, V, L-Cuneus]	93.36±1.56	74.22±1.01	95.30±0.32	84.73	66.55	79.27
5	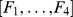	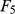 : [119, SA, R-Parsopercularis]	91.98±1.32	75.43±1.01	95.30±0.86	82.91	69.27	81.09
6	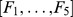	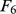 : [84, SA, L-Paracentral]	94.90±0.78	81.46±0.84	98.94±0.17	82.00	74.73	79.27
7	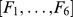	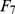 : [227, FI, L-Precentral]	96.36±1.28	85.43±0.96	88.57±0.87	85.64	78.36	82.91
8	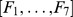	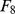 : [272, FI, R-Insula]	96.18±1.00	85.13±0.50	88.84±0.79	88.36	81.09	82.91
9	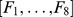	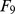 : [148, V, L-Lingual]	95.64±1.23	84.83±0.84	89.45±0.99	88.36	79.27	81.09
10	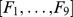	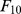 : [218, FI, L-Middletemporal]	97.51±1.83	89.35±1.00	98.94±0.89	87.45	80.18	85.64
11	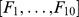	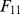 : [101, SA, L-Transversetemporal]	97.49±1.03	87.87±1.46	96.18±0.82	**90.18**	78.36	83.82
12	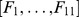	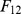 : [264, FI, R-Rostralmiddlefrontal]	96.66±0.83	88.74±1.36	97.54±0.99	88.36	80.18	82.00
13	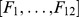	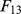 : [225, FI, L-Postcentral]	96.42±1.21	88.45±1.05	95.34±0.81	87.45	79.27	80.18
14	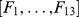	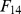 : [265, FI, R-Superiorfrontal]	96.31±1.17	88.81±0.87	92.23±1.01	87.45	81.09	82.00
15	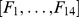	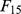 : [131, SA, R-Superiortemporal]	96.46±1.05	91.65±0.75	92.15±0.87	88.36	**84.73**	83.82
16	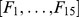	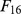 : [252, FI, R-Middletemporal]	97.04±0.88	92.58±1.13	94.58±0.97	86.55	83.82	83.82
17	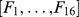	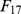 : [80, SA, L-Lingual]	97.26±0.99	92.06±1.22	94.47±0.84	87.45	84.73	85.64
18	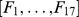	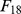 : [122, SA, R-Pericalcarine]	97.86±0.82	92.77±1.00	95.22±0.82	87.45	84.73	85.64
19	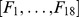	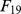 : [204, V, R-Insula]	98.30±0.94	92.96±0.99	98.46±0.62	88.36	83.82	**86.55**

ED: Experimental Dataset; SA: Surface Area; V: Volume; FI: Folding Index; L: Left; R: Right.

**Figure 5:**
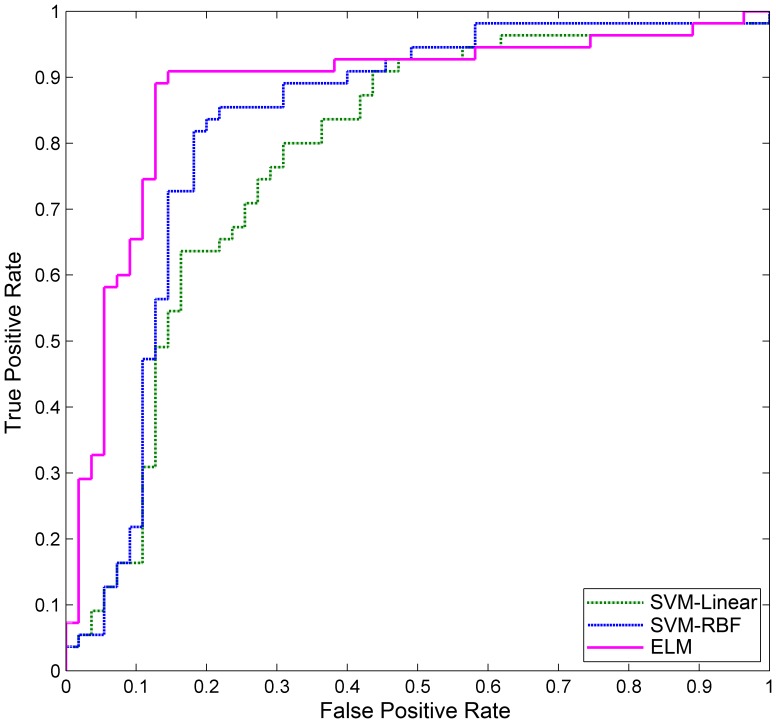
The receiver operating characteristics (ROC) curve for three classifiers discriminating between ADHD patients and healthy controls.

To further compare the three methods, the receiver operating characteristics (ROC) curves were generated by varying a threshold applied to the continuous prediction score that each of the algorithms generated (Figure 5). The area under the ROC curve (AUC) for ELM is 0.8757, for SVM-Linear is 0.7792, and for SVM-RBF is 0.8258. Therefore, ELM performs the best for discriminating ADHD patients from healthy controls.

### 3. Permutation Tests for ELM

**Figure 6:**
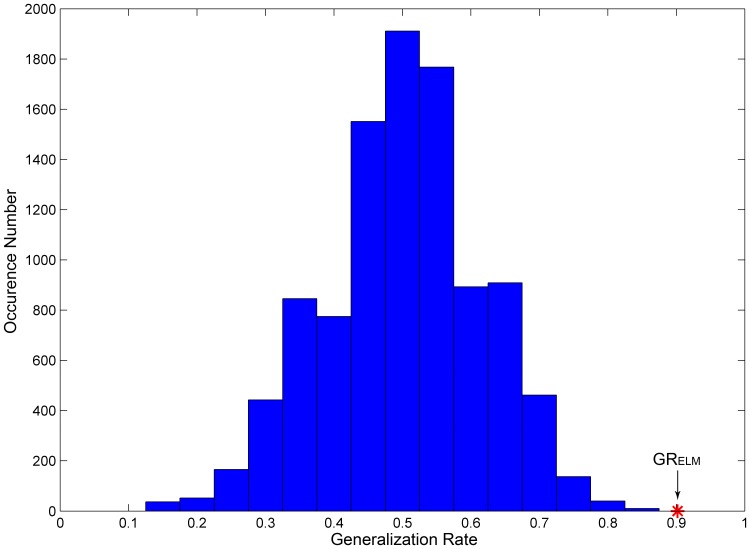
The permutation distribution of the estimate using the ELM classifier. X-label and y-label respectively represent the generalization rate and occurrence number. 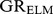 refers to the generation rate obtained by training on the real class labels.

The permutation distribution of the estimate using the ELM classifier is shown in Figure 6. With the generalization rate as the statistic, cross-validation was performed on the 11 most discriminating features and the permutation test was repeated for 10000 times. This figure indicate that the ELM classifier learned the relationship between the data and the labels with a probability of being wrong of 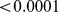.

## Discussion

In this study, we established an automatic and efficient ADHD classification method using the ELM learning algorithm on structural MRI data to provide accurate, objective clinical diagnosis. In this study, we achieved two main findings. First, our results indicate that it is possible to classify ADHD and control subjects with a high degree of accuracy using an automatic procedure that combines structure with ELM. Our results from ADHD and control classification achieved an excellent prediction accuracy of 90.18%. This high testing accuracy will improve the actual auxiliary diagnostic accuracy. Second, we demonstrated that the ELM method is much faster (more than 1000 times faster) than other prediction models, such as SVM, making the ELM algorithm a high efficiency method for ADHD diagnosis.

### 1. Efficient Brain Structure Features in ADHD Classification

The cortex can be divided into five major segments according to the anatomical structure and function of the human brain, including the frontal lobe, the occipital lobe, the parietal lobe, the temporal lobe and the cingulate. To further understand the relationship between different brain segments and the etiology of ADHD, we pick off the most discriminative 11 brain structure features from the classification results and categorize them in major lobes shown in Table 4.

**Table 4:** Most discriminative brain structure features for ADHD classification.

Lobe	Segmentation	Feature
Frontal	R- Pars Opercularis	SA
	L- Paracentral	SA,FI
Temporal	L- & R- Transverse Temporal	SA,FI
	L- Middle Temporal	FI
Occipital	L- & R- Cuneus	SA,V
	L- Lingual	V
Insular	L- & R- Insular	FI

The cuneus and lingual are portions of the human brain in the occipital lobe. Both of them are linked to receiving and processing the visual information, especially related to letters. The disorder of these portions of brain can lead to a confusion of visual information which may further cause inattention. Additionally, insular cortex is a portion of the cerebral cortex folded deep within the lateral sulcus separating the temporal lobe from frontal lobes. Numerous studies have established that frontal lobe, temporal lobe and insular are mainly associated with attention, motivation, sensory, emotions and memory, which are likely to be involved in ADHD behavioral symptoms, such as inattention, hyperactivity, impulsivity and impaired executive function. In addition, since the ELM classification relied heavily on the anatomical MRI data of these regions, these findings could indicate that these cortical regions mentioned above have the most ADHD-related structural changes in the human brain.

### 2. Computational Efficiency of ADHD Classification

The computational efficiency of a pattern recognition method directly influences the performance of ADHD diagnosis in practice. An ideal ADHD machine classification method should achieve both high discrimination accuracy and fast classification speed. In the data presented in Figure 3, the ADHD classification time of the ELM was significant lower than both SVM algorithms. This may be due to that the SVM algorithm requires several user decisions, including the choice of the kernel parameters 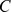 and 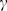, which usually take plenty of extra training time. In contrast, the ELM learning algorithm chooses hidden nodes randomly and determines the output weights of the feedforward neural networks analytically by calculating the Moore-Penrose generalized inverse 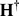 of the hidden layer output matrix 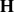. This has important implications. In particular, it indicates that there is no need for the ELM algorithm to spend extra training time on parameter searches and nearly unaffected by changing of experimental dataset size. Another major contribution to our ADHD classifier came from the relatively high classification accuracy (achieved a maximum prediction accuracy of 90.18%). All of these suggest that ELM achieves higher computing efficiency than SVM and make it possible for the ELM learning algorithm to be efficiently applied to ADHD classification. It is also worth noting that, although ELM algorithm performs better in generalization compared with conventional learning methods, too much hidden layer nodes chosen may lead to overfitting and impact the performance in practical application. Therefore, it is essential to determine the optimal number of nodes before training to avoid overfitting.

### 3. Influence of Subject Sample Sizes

**Figure 7:**
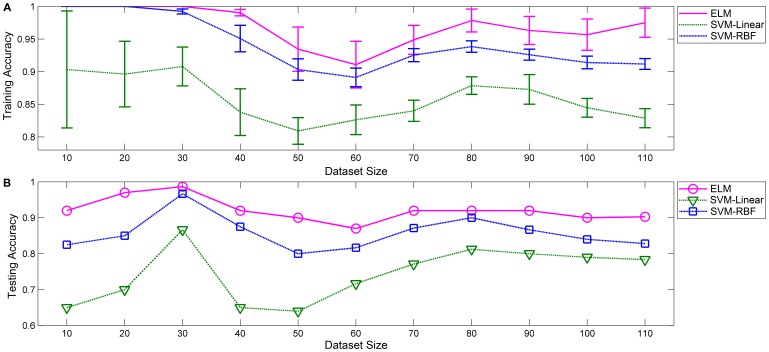
Classification accuracy of ADHD using ELM, SVM-Linear and SVM-RBF with different experimental dataset sizes. The results are calculated using different experimental dataset sizes (from 10 to 110). (A) Training accuracy for three algorithms with different experimental dataset sizes. (B) Testing accuracy for three algorithms with different experimental dataset sizes.

For traditional pattern recognition methods, a large training sample is usually necessary to ensure classification accuracy because most common pattern recognition algorithms are probabilistic and use statistical inference to determine the best label for a given instance [41]–[44]. For example, several recent reports have demonstrated good performance in AD classification using different modalities of features. One of the common practices in these previous studies is the utilization of hundreds of training samples to achieve better classification accuracy [44]–[47]. The dependency of a classifier on training sample size is also an important criterion for evaluating the performance of a classifier. To further compare the ADHD classification performance of ELM and SVM for different experimental dataset sizes, we randomly extracted and combined data from all 110 subjects preprocessed MRI datasets into eleven new experimental datasets respectively containing 10, 20, 30, 40, 50, 60, 70, 80, 90, 100 and 110 subjects. Each new experimental dataset consists of half ADHD subjects and half healthy controls. All three algorithms were used to evaluate the eleven ADHD experimental datasets. The results are shown in Figure 7.

The average training and testing rates of three methods are all influenced to a certain extent by the experimental dataset size, while the overall ADHD classification accuracy of ELM is significantly higher than that of both SVM algorithms during the whole experiment process. In contrast to SVM, the ELM algorithm performs more smoothly in ADHD discrimination with the changing of experimental dataset size (Figure 7B). This suggests that ELM algorithm has a higher robustness and adaptability on different experimental dataset size. Together with advanced feature selection methods, ELM is likely to be a powerful imaging-based pattern recognition method for ADHD diagnosis.

### 4. Effect of Medication

In our study, thirty of 55 adolescents with ADHD received medical treatment. For ADHD medication, stimulant medications are the most frequently choice of pharmaceutical treatment. There are a number of non-stimulant medications, such as atomoxetine, that may be used as alternatives [48]. Some research show that patients with attention deficit hyperactivity disorder (ADHD) and a medication history present abnormal brain activation in prefrontal and striatal brain regions during cognitive challenge. Atomoxetine improved inhibitory control and increased activation in the right inferior frontal gyrus [49], [50]. This may caused by atomoxetine increased extracellular (EX) concentrations of norepinephrine and dopamine in prefrontal cortex [51]. However, to the best of our knowledge, there is a lack of evidence on medication effects on changing the brain structure of the ADHD patients. Additionally, psychostimulant medications were withheld at least 48 hours prior to scanning in our study. Therefore, we ignored the influence of drugs on brain structure changing in the current study. More work and investigations will be needed to understand the influence of ADHD medication in the future study.

### 5. Limitations

The current study only considers structural MRI data from the subjects in the ADHD-200 Global Competition. Several resting state functional connectivity studies suggest that ADHD is associated with large-scale brain sub-networks dysfunction [52], [53]. In the future, we will use additional modalities (i.e., fMRI, PET and DTI) with our current classification method to further improve ADHD classification performance. Moreover, since classification accuracy was directly impacted by the selected features, an efficient feature selection method may greatly improve the performance of a learning algorithm. In our current study, conventional feature selection methods, F-Score and SFS, were combined to obtain the optimizing classification features. This method, as simply based on geometry theory, can effectively select the optimizing features. However, it cannot consider the interrelationships among different patterns of data when classifying using multiple modalities data. Sparse representation, one of the latest feature selection methods, has been recently demonstrated to be an efficient feature selection method in pattern recognition of structural MRI scans [54]. It has become popularity since its ability to contrast high dimensional data with compressed samples especially in multivariate pattern analysis. Therefore, we will utilize the advanced sparse representation method combining with multiple modal data and efficient learning methods for ADHD classification in the future.

## Conclusion

To our knowledge, this is the first study to propose an ADHD classification model using the extreme learning machine (ELM) with F-score and SFS feature selection methods to perform objective diagnosis. Our results show that the ELM algorithm has considerably good performance and an extremely high efficiency in discriminating ADHD subjects from healthy controls. Compared with traditional ADHD diagnosis methods, ELM has the following advantages: 1. extremely fast discrimination speed and satisfactory high classification accuracy; 2. ADHD discrimination using objective MRI data; 3. excellent ADHD classification performance with small training sample sizes and robustness with changes in sample size and 4. does not need to select the training parameters because the hidden nodes are randomly chosen. Moreover, we observed that the frontal lobe, temporal lobe, occipital lobe and insular are potentially involved in ADHD-related structural changes in the human brain. These findings suggest that our ADHD classification method using the ELM learning algorithm is not only a promising method for ADHD aided diagnosis and the study of disease etiology but can also identify which features of the brain are involved in different diseases.

## Supporting Information

Appendix S1 (DOC)
