# Supplementary material for: Extreme Learning Machine-Based Classification of ADHD Using Brain Structural MRI Data
Source: PLoS One. 2013 Nov 19;8(11):e79476. doi: 10.1371/journal.pone.0079476 (PMC3834213; doi:10.1371/journal.pone.0079476)
Supplement: Appendix S1 — (DOC) [file pone.0079476.s001.doc]

**Appendix S1**

**Theorem 1.** *Given a standard SLFN with N hidden nodes and activation function g : $R\to R$ which is infinitely differentiable in any interval, for N arbitrary distinct samples $\left(\mathbf{x}_{i},\mathbf{t}_{i}\right)$, where $\mathbf{x}_{i}\in\mathbf{R}^{n}$ and $\mathbf{t}_{i}\in\mathbf{R}^{m}$, for any $\mathbf{w}_{i}$ and $b_{i}$ randomly chosen from any intervals of $\mathbf{R}^{n}$ and $\mathbf{R}$, respectively, according to any continuous probability distribution, then with probability one, the hidden layer output matrix $\mathbf{H}$ of the SLFN is invertible and* $\left\|\mathbf{H}\beta-\mathbf{T}\right\|=0$.

**Proof.** Let us consider a vector $\mathbf{c}\left(b_{i}\right)=\left[g_{i}\left(\mathbf{x}_{1}\right),\ldots,g_{i}\left(\mathbf{x}_{N}\right)\right]^{\text{T}}=\left[g\left(\mathbf{w}_{i}\cdot\mathbf{x}_{1}+b_{i}\right),\ldots,g\left(\mathbf{w}_{i}\cdot\mathbf{x}_{N}+b_{i}\right)\right]^{\text{T}}$, the $i$th column of $\mathbf{H}$, in Euclidean space $\mathbf{R}^{N}$, where $b_{i}\in\left(a,b\right)$ and $\left(a,b\right)$ is any interval of $\mathbf{R}$.

**Theorem 2.** *Given any small positive value $\varepsilon>0$ and activation function g : $R\to R$ which is infinitely differentiable in any interval, there exists $\tilde{N}\leq N$ such that for N arbitrary distinct samples $\left(\mathbf{x}_{i},\mathbf{t}_{i}\right)$, where $\mathbf{x}_{i}\in\mathbf{R}^{n}$ and $\mathbf{t}_{i}\in\mathbf{R}^{m}$, for any $\mathbf{w}_{i}$ and $b_{i}$ randomly chosen from any intervals of $\mathbf{R}^{n}$ and $\mathbf{R}$, respectively, according to any continuous probability distribution, then with probability one, $\left\|\mathbf{H}_{N\times\tilde{N}}\beta_{\tilde{N}\times m}-\mathbf{T}_{N\times m}\right\|<\varepsilon$.*

**Proof.** The validity of the theorem is obvious, otherwise, one could simply choose $\tilde{N}=N$ which makes $\left\|\mathbf{H}_{N\times\tilde{N}}\beta_{\tilde{N}\times m}-\mathbf{T}_{N\times m}\right\|<\varepsilon$ according to Theorem 1.

**Theorem 3.** *Let there exist a matrix $\mathbf{G}$ such that $\mathbf{Gy}$ is a minimum norm least-squares solution of a linear system $\mathbf{Ax}=\mathbf{y}$. Then it is necessary and sufficient that $\mathbf{G}=\mathbf{A}^{\dagger}$, the Moore–Penrose generalized inverse of matrix $\mathbf{A}$.*
